# Epidermal growth factor and its influencing variables in healthy children and adults

**DOI:** 10.1371/journal.pone.0211212

**Published:** 2019-01-24

**Authors:** Sarang Meybosch, Amandine De Monie, Charlotte Anné, Luc Bruyndonckx, Angelika Jürgens, Benedicte Y. De Winter, Dominique Trouet, Kristien J. Ledeganck

**Affiliations:** 1 Laboratory of experimental medicine and pediatrics, University of Antwerp, Antwerp, Belgium; 2 Department of pediatric nephrology, Antwerp University Hospital, Antwerp, Belgium; International University of Health and Welfare, School of Medicine, JAPAN

## Abstract

**Background & objective:**

Epidermal growth factor (EGF) stimulates cell proliferation and differentiation after binding to its receptor. Next to its role in magnesium homeostasis, EGF disturbances have been described in oncology, diabetes and autism spectrum disorders. The aim of this study was to determine EGF serum and urine values for both healthy children and adults. Next, we investigated the relation between several variables and urinary and serum EGF concentrations.

**Methods:**

Both healthy adults (n = 50) and children (n = 78) were included. Serum and urinary EGF concentrations were measured with ELISA technology.

**Results:**

Serum EGF was inversely correlated with age (r = —0.873; p<0.001) and positively correlated with serum magnesium (r = 0.597; p<0.001). The urinary EGF was also inversely correlated with age (r = -0.855; p<0.001). In adults and children older than 13 years of age, the urinary EGF significantly differed between sexes (p = 0.001). Urinary EGF was positively correlated with serum magnesium (r = 0.583; p<0.001) and creatinine clearance (r = 0.524; p<0.001) and negatively correlated with the fractional excretion of magnesium (r = 0.248; p = 0.014). In a multivariate model, age and serum magnesium remained independently related to serum EGF while age, serum EGF and serum magnesium remained independently related to urinary EGF.

**Conclusions:**

This study provides valuable insights in urinary and serum EGF patterns in healthy subjects. By systematically correcting EGF for body surface, significant correlations with age, gender and magnesium were observed.

## Introduction

Human epidermal growth factor (EGF), a 6.000 molecular weight polypeptide, was first isolated by Cohen and Carpenter in 1975 [1]. EGF is a growth factor that stimulates cell growth, proliferation and differentiation by binding to its receptor EGFR [2]. It has been proven to be a potent mitogen by stimulating mRNA, DNA and protein synthesis of epithelial cells [3]. EGF is locally produced in several tissues, such as Henle’s loop and the distal convoluted tubule in the kidney, salivary glands and duodenum [4].

In the kidney, EGF is involved in the repairing process of renal tissues [3, 5]. In an animal model of acute renal failure, EGF proved to be a potent growth promoter by regenerating renal tubular cells and accelerating the recovery of a normal renal function [6, 7]. Moreover, EGF is highly expressed along the distal convoluted tubule (DCT), which is an important site for regulating urinary magnesium excretion and thus magnesium homeostasis. It stimulates magnesium reabsorption by the transient receptor potential cation channel 6 (TRMP6), located at the apical membrane of the DCT cells [8–10]. Our own research group demonstrated that EGF is involved in the pathophysiology of drug-induced renal magnesium loss [11–13].

High concentrations of EGF can be found in the urine. Based on in vitro experiments, it has been previously suggested that urinary EGF originates from the ultrafiltrate. However, in vivo, it was shown in rats and in humans that the urinary EGF is mainly produced in the kidney itself. Therefore, it is generally accepted that the urinary EGF excretion reflects the renal EGF production [3, 14–17]. Reduced concentrations of EGF in the urine have been previously observed in diabetes nephropathy, IgA nephropathy, adult polycystic kidney disease, and children with chronic renal failure. Also, the possibility that urinary EGF might serve as a surrogate marker for functional regeneration of the renal tubules, reflecting their ability to respond to future acute or chronic injury was recently put forward [18].

In addition to its role in magnesium homeostasis, EGF disturbances have been described in patients suffering from oncological pathologies [19], diabetes [20] and autism spectrum disorders [21]. A study in diabetic patients showed that the excretion of EGF was independently and inversely correlated with age and duration of diabetes [20]. Epidermal growth factor is furthermore involved in the proliferation and growth of neurons and glia of the central nervous system (CNS). High levels of EGF are present in the CNS and play a critical role in controlling proliferation and differentiation of the nervous tissue during neurogenesis [22, 23]. In this context, Onore et al. found that levels of plasma EGF were significantly reduced in young children with autism spectrum disorder when compared with age-matched control children [21].

Considering the importance of EGF in magnesium homeostasis, its oncological properties, and its role in diabetes and autism, it is important to further investigate the serum and urinary EGF reference values in healthy subjects and the possible variables influencing these values.

A few studies reported on urinary EGF values in either children [5, 24] or adults [19] and 1 study included both [3]. From these studies, the urinary EGF concentration appeared to decrease with age. None of these studies, however, extensively investigated other variables such as gender, length or renal function related to urinary EGF concentrations. As EGF concentrations seem to be related to age, differences between children and adults should be investigated more in depth.

Serum EGF values in healthy subjects have been reported in 1 study [25], however, without going into detail on relating variables. Biological differences in serum EGF concentrations thus still need to be explored in healthy children and adults to gain more insight in normal EGF concentrations and associating variables before one can further investigate its role in pathophysiology on a clinically standardized basis.

The aim of this study was to determine serum and urinary EGF values in both healthy children and adults. Next, we investigated the relation between different variables such as age, gender, urinary and serum magnesium.

## Subjects and methods

### Study design and patients

The study was conducted in accordance with the Declaration of Helsinki and the principles of Good Clinical Practice. The study protocol was approved by the Ethics Committee of the Antwerp University Hospital (file numbers 11/5/51 (adults) and 9/44/231 (children)). All patients and the parents or legal guardians of the children below 18 years old gave a written informed consent.

#### Adults

The adults (n = 50) were all healthy volunteers, who were recruited at the University of Antwerp. From each of these patients a single blood and urine sample was collected. Each adult completed a questionnaire on medical history and age and declared not to be on any medication.

#### Children

The children (n = 78) were recruited in two ways. Forty-four of them were enrolled at the sleep lab at the Antwerp University Hospital and were considered healthy after a normal polysomnography and normal clinical investigation. Thirty-four children were recruited voluntary by email advertisement in our faculty. From 49 children both a single blood and urine sample was collected. From 29 children a single urine sample was collected. The children’s parents completed a questionnaire on medical history and biometrics. None of the children was taking any chronic medication.

#### Exclusion criteria

Patients with a chronic medical condition or on any chronic medication were excluded.

### Clinical data

#### Body surface area

In children, the body surface area was calculated from length and weight as defined by Mosteller [26]: BSA (m^2^) = (height (cm) x weight (kg)/3600)^½^.

#### Body mass index (BMI)

The body mass index was calculated as follows: weight (kg)/length (m)^2^

#### Z-scores

For children, Z-scores were calculated for BMI, length and weight.

#### Age categories

In order to investigate the EGF concentrations according to age, we divided the population into 7 age categories as follows: 3–6 years old; 7 to 12 years old; 13 to 15 years old; 16 to 18 years old, 23 to 30 years old, 30–50 years old and > 50 years old.

### Laboratory measurements

#### Determination of creatinine and magnesium

Serum and urine creatinine and magnesium were analysed with the Dimension Vista system (Siemens Healthcare Diagnostics, Deerfield, USA) using an ECREA or Mg flex reagent cartridge respectively.

Creatinine clearance was calculated using the Bedside Schwartz equation, which is the recommended equation to estimate glomerular filtration rate (GFR) in children [27]. In adults, creatinine clearance was calculated by use of the CKD-EPI formula, as recommended by KDIGO guidelines for creatinine clearance > 60 ml/min/1.73m^2^ [28].

#### Determination of urinary and serum EGF

Serum and urinary EGF were measured using an EGF human Elisa kit (Invitrogen, California, USA), according to the manufacturer’s guidelines. The detection limit of this assay was 3.9 pg/ml. A preliminary experiment (n = 10) was performed to test the intra- and inter-variability of the EGF human Elisa kit, showing a mean intra-assay coefficient of variance of 9.82% and an inter-assay coefficient of variation of 9.81%.

Urinary EGF was corrected for urinary creatinine, in order to correct for differences in urine concentration, analogous to previous studies [3, 5]. The correction for urinary creatinine is expressed as “uCr”.

### Statistical analysis

The statistical analysis was performed using IBM SPSS 24 Statistics. Normality of the different variables was analysed by the Kolmogorov-Smirnov test. Correlation between two variables was determined by Spearman’s correlation test in case one of both was skewed and with the Pearson correlation test when both variables were normally distributed. The results of serum and urinary EGF corresponding with specific age categories are expressed as median, with minimum and maximum. A p-value below 0.05 was considered statistically significant. A multivariate linear regression analysis for serum and urinary EGF was performed. All variables that were significantly correlated with the dependent variable (serum or urinary EGF) were included into the multivariate model. In the multivariate models, EGF was analysed in its absolute form as the dependent variable to avoid spurious correlations with other variables that were included in the model such as EGF, creatinine clearance or FE Mg^2+^.

## Results

### Study population

The study population consisted of 128 Belgian healthy subjects in total, of which 51 men and 77 women. The age ranged from 3 to 74 years, with a median age of 16 years.

#### Adults

The adult population (n = 50) consisted of 16 men and 34 women. The age ranged from 23 years old to 74 years old, with a median age of 32 years.

#### Children

Seventy-eight children were included, ranging from 3 to 18 years old. Thirty-five of them were boys, 43 were girls. Median weight was 38 kg (14–94 kg) and the mean weight Z-score was 0.25 ± 1.09. The median length was 1.47 m (0.94–1.81 m) and the mean length Z-score was -1.18 ± 1.19. The median BMI was 18 kg/m^2^ (12–31 kg/m^2^) and the mean body mass index Z-score was -0.05 ± 1.15.

### EGF concentrations in serum and urine

Table 1 depicts the serum and urinary EGF concentrations in the entire population and in adults and in children separately.

**Table 1:** Serum and urinary EGF concentrations in the entire population, in adults and in children.

	Entire population	Children	Adults
**Serum EGF (pg/ml)**	368 (2–2087)	1070 (200–2087)	158 (2–381)
**Serum EGF/BSA (pg/ml/1.73m^2^)**	368 (2–5518)	1310 (314–5518)	158 (2–381)
**Urinary EGF (ng/ml)**	49 (1–219)	67 (15–219)	14 (1–73)
**Urinary EGF/uCr (ng/mg Cr)**	30 (4–173)	56 (23–173)	18 (4–41)
**Urinary EGF/uCr/BSA (ng/mg Cr/1.73m^2^)**	54 (4–424)	68 (21–424)	18 (4–41)

BSA: body surface area; uCr: urinary creatinine.

Both urinary and serum EGF concentrations depend on age, so a subanalysis was performed to determine EGF concentrations in the different age categories. The age specific EGF concentrations are presented in Table 2 (serum) and Table 3 (urine).

**Table 2:** Age-specific serum EGF concentrations.

Age category	Serum EGF (pg/ml)	Serum EGF/BSA (pg/ml/1,73m^2^)
**≤ 7 years (n = 14)**	1265 (684–2087)	2463 (1432–5518)
**7–13 years (n = 16)**	1020 (200–1569)	1344 (314–1995)
**13–16 years (n = 11)**	1073 (467–1611)	1177 (595–1392)
**16–18 years (n = 10)**	984 (417–1425)	1068 (426–1498)
**23–30 years (n = 18)**	210 (7–381)	210 (7–381)
**30–50 years (n = 16)**	209 (2–314)	209 (2–314)
**≥ 50 years old (n = 16)**	76 (17–337)	76 (17–337)

Absolute serum EGF concentrations and EGF concentrations corrected for body surface are displayed. EGF: epidermal growth factor; BSA: body surface area.

**Table 3:** Age-specific urinary EGF concentrations.

Age category	Urinary EGF (ng/ml)	Urinary EGF/uCr (ng/mg Cr)	Urinary EGF/uCr/BSA (ng/mg Cr/1,73m^2^)
**≤ 7 years (n = 21)**	96 (23–219)	107 (55–173)	237 (125–424)
**7–13 years (n = 27)**	68 (15–136)	58 (24–102)	83 (29–129)
**13–16 years (n = 18)**	52 (20–176)	39 (26–84)	42 (25–76)
**16–18 years (n = 12)**	63 (18–112)	37 (23–64)	38 (21–67)
**23–30 years (n = 18)**	14 (2–73)	25 (8–41)	25 (8–41)
**30–50 years (n = 16)**	17 (1–50)	16 (4–38)	16 (4–38)
**≥ 50 years old (n = 16)**	9 (3–40)	15 (5–33)	15 (5–33)

Absolute urinary EGF concentrations, urinary EGF concentrations corrected for urinary creatinine concentration and corrected for creatinine and body surface are displayed. EGF: epidermal growth factor; uCr: urinary creatinine concentration; BSA: body surface area.

### Correlation analysis

#### Serum EGF

**Fig 1:**
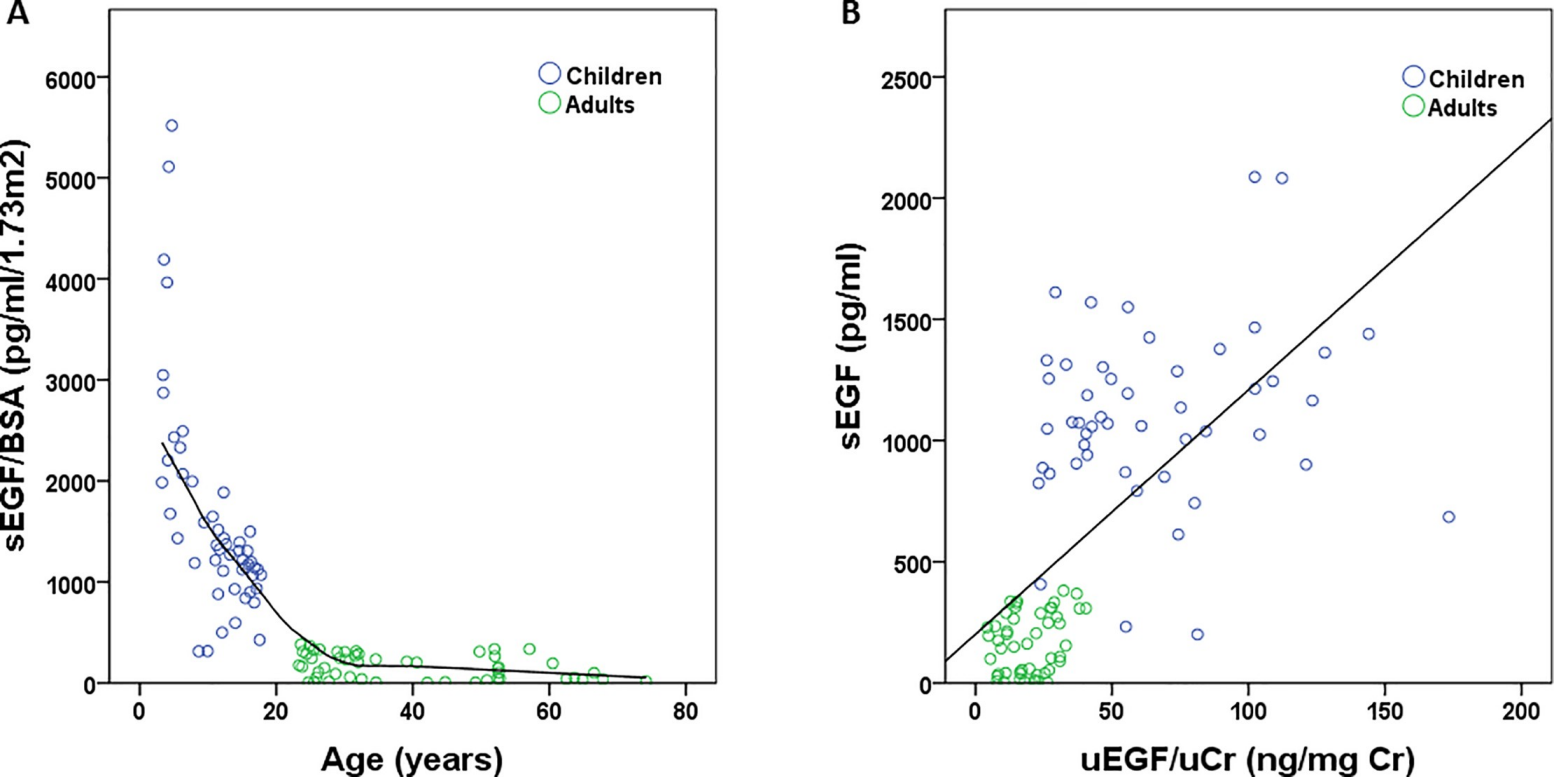
Correlations of serum EGF with age (A; r = -0.873; p<0.001) and with urinary EGF (B; r = 0.716; p<0.001). EGF was not corrected for body surface to avoid spurious correlation. sEGF: serum epidermal growth factor; uEGF: urinary epidermal growth factor; uCr: urinary creatinine; BSA: body surface area.

Age and length were both significantly and inversely correlated with serum EGF/BSA (r = -0.873; p<0.001 and r = -0.685; p<0.001 respectively) as depicted in Fig 1A. Based on these significant correlations, we further decided to correct the EGF concentration (both serum and urine) for body surface when comparing adults with children. We hereby define the correction for body surface area as “BSA”. Serum EGF and urinary EGF/uCr were significantly and positively correlated (r = 0.716; p<0.001), as shown in Fig 1B. Sex was not significantly correlated with serum EGF/BSA (p = 0.535).

#### Urinary EGF

**Fig 2:**
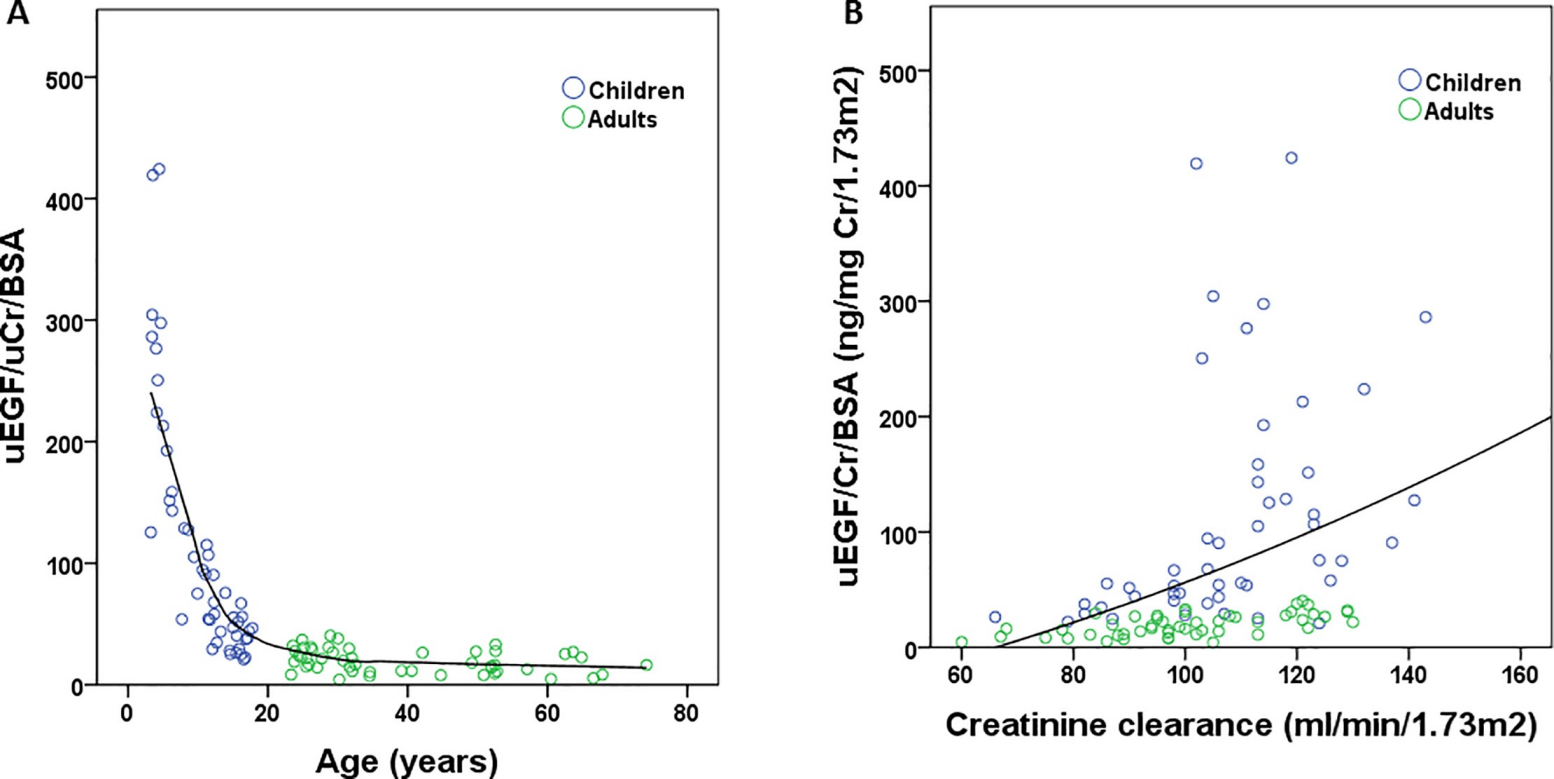
Correlations of urinary EGF with age (A; r = -0.855; p<0.001) and with creatinine clearance (B; r = 0.524; p<0.001). uEGF: urinary epidermal growth factor; uCr: urinary creatinine; BSA: body surface area.

Urinary EGF/uCr/BSA was significantly correlated with both age and length (r = -0.855; p<0.001; Fig 2A and r = -0.893; p<0.001 respectively).

**Fig 3:**
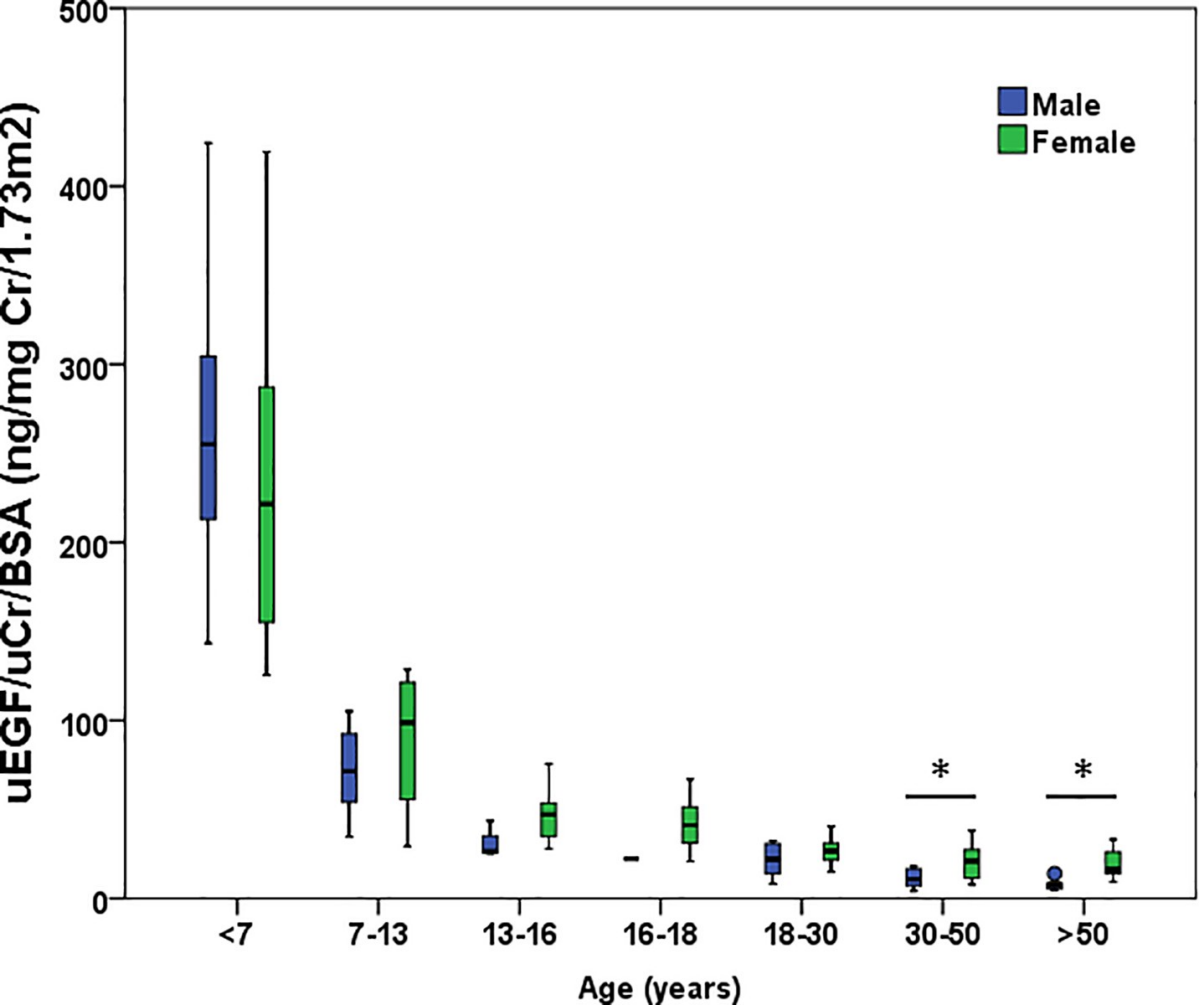
Serum EGF concentrations between sexes per age subcategory. In females >13y, urinary EGF is significantly higher compared to males. * p<0.05. uEGF: urinary epidermal growth factor; uCr: urinary creatinine; BSA: body surface area.

In the whole study population, urinary EGF/uCr/BSA and sex did not significantly correlate (p = 0.408). As we believed that puberty and the accompanying change in hormonal status could influence EGF values, we re-analysed the data with a cut-off point of 13y. When analysing the effect of gender on urinary EGF from that age on, we found a significant correlation. The urinary EGF significantly differed between sexes (p = 0.001) with a median urinary EGF/uCr/BSA value of 14.00 ng/mg Cr/1.73m2 (4.27–43.70 ng/mg Cr/1.73m2) in men and 26.95 ng/mg Cr/1.73m2 (7.79–75.56 ng/mg Cr/1.73m2) in women. Next, we repeated the analysis for the cut-off point of 12y and the effect of gender disappeared. We thus concluded that 13y was the correct cut-off point for an effect of gender on urinary EGF values. When further analysing age subcategories in patients older than 13y, the differences in urinary EGF between males and females remained significantly different in age categories 30-50y (p = 0.031) and in patients older than 50y (p = 0.003; as presented in Fig 3). Of note, the number of patients per subcategory was rather small (n = 9–18 per age subcategory), which might explain why not all differences remained significant. The urinary EGF/uCr/BSA values in females from 13 to 18 years old were significantly higher than the values in females older than 18 years of age (p<0.001). The same was true for males of 13 to 18 years old compared to the males older than 18 years old (p = 0.011).

As shown in Fig 2B, creatinine clearance significantly and positively correlated with urinary EGF/uCr/BSA (r = 0.524; p<0.001).

### Relation between EGF and the magnesium homeostasis in healthy subjects

#### Serum EGF

**Fig 4:**
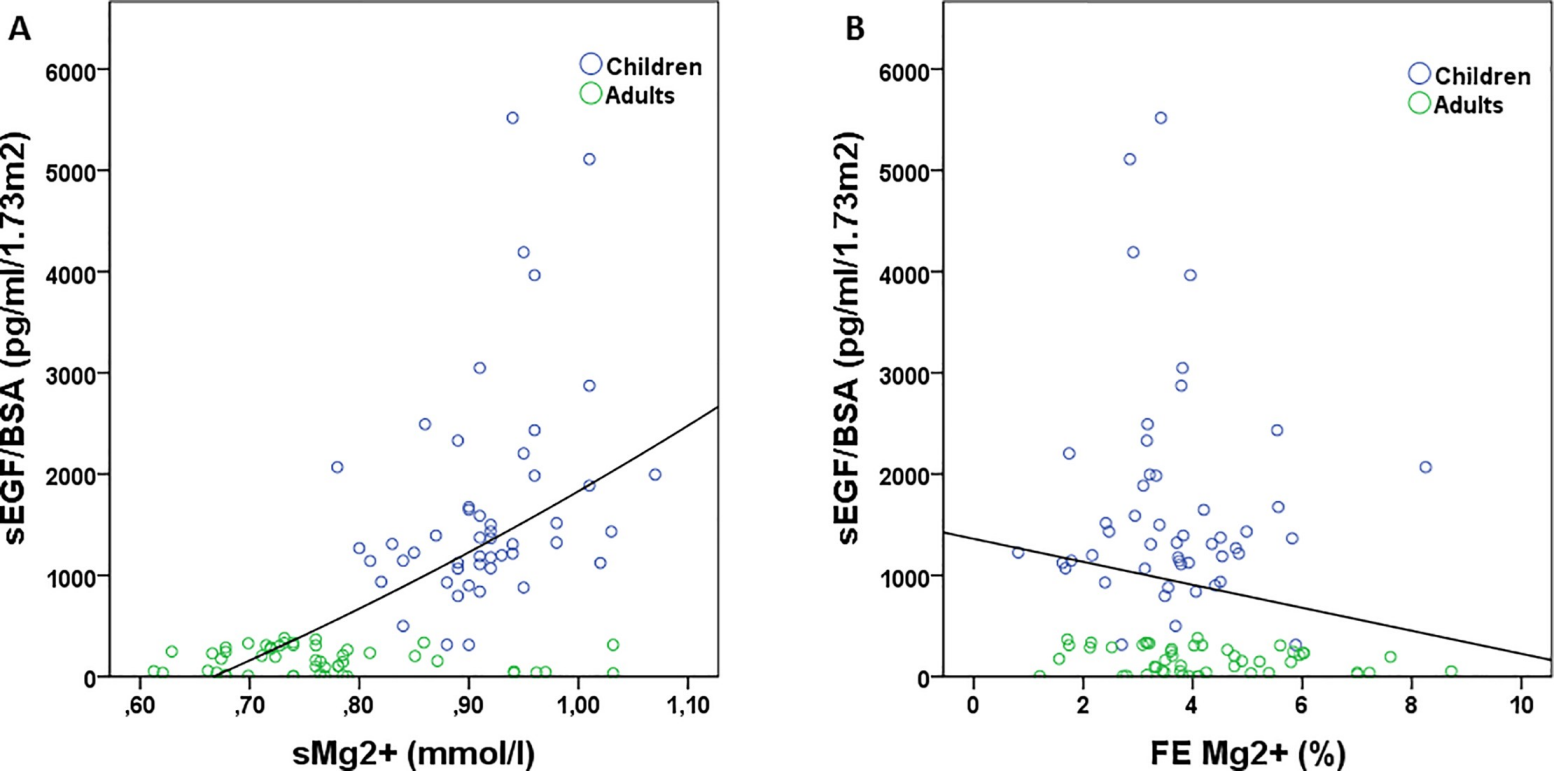
Correlation between serum EGF and serum magnesium (A; r = 0.597; p<0.001) and serum EGF and the FE Mg^2+^ (B; r = -0.200; p = 0.048). sEGF: serum epidermal growth factor; BSA: body surface area; sMg2+: serum magnesium; FE Mg^2+^: fractional excretion of magnesium.

Serum EGF/BSA and serum magnesium concentration were significantly and positively correlated (r = 0.597; p<0.001; Fig 4). Serum EGF/BSA and fractional excretion of magnesium were significantly and negatively correlated (r = -0.200; p = 0.048).

#### Urinary EGF

**Fig 5:**
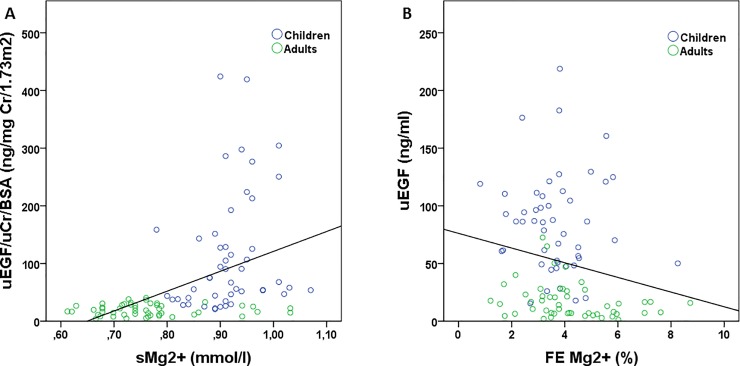
Correlation between urinary EGF and serum magnesium (A; r = 0.583; p<0.001) and urinary EGF and the FE Mg^2+^ (B; r = -0.248; p = 0.014). uEGF: urinary epidermal growth factor; uCr: urinary creatinine; BSA: body surface area; sMg2+: serum magnesium; FE Mg^2+^: fractional excretion of magnesium.

Serum magnesium and urinary EGF/uCr/BSA were significantly and positively correlated (r = 0.583; p<0.001; Fig 5A) while urinary EGF concentration and fractional excretion of magnesium were significantly and inversely correlated (r = -0.248; p = 0.014; Fig 5B).

### Variables independently related to EGF

Table 4 depicts a summary of all the variables in a univariate correlation, with the corresponding statistical findings.

**Table 4:** Summary of the univariate correlation analysis, depicting the different variables in relation to serum and urinary EGF concentrations.

	Serum EGF/BSA	Serum EGF	Urinary EGF/uCr/BSA	Urinary EGF
**Age**	R = -0.873; p<0.001		R = -0.855; p<0.001	
**Length**	R = -0.685; p<0.001		R = -0.893; p<0.001	
**Urinary EGF/uCr**		R = 0.716; p<0.001		
**Gender > 13y (1 = F)**	P = 0.317		R = 0.422; p<0.001	
**Creatinine clearance**			R = 0.524; p<0.001	
**Serum Mg2+**	R = 0.597; p<0.001		R = 0.583; p<0.001	
**FE Mg^2+^**	R = -0.200; p = 0.048			R = -0.248; p = 0.014

EGF: epidermal growth factor; BSA: body surface area; uCr: urinary creatinine; FE Mg2+: fractional excretion of magnesium; F: female; r: correlation coefficient; p: level of significance.

In order to investigate the variables that independently correlate with EGF, the variables that significantly correlated with EGF were tested in a multivariate model. The 99 patients with both a serum and urine sample were included in the multivariate analysis.

#### Serum EGF

To determine independent variables related to serum EGF, age, urinary EGF/UCr, serum magnesium and the fractional excretion of magnesium were analysed in a multivariate model. As serum EGF was skewed, a logarithmic transformation was performed and the log serum EGF was tested as the dependent variable. Length was not included in the multivariate model since data on length were only available in children. Age and serum Mg^2+^ remained significant and independent predictors of log serum EGF as shown in Table 5. The model declares 54.2% of the variance in log serum EGF.

**Table 5:** Independent variables related to serum EGF.

	B	95% confidence interval		p-value
		Lower	Upper	
**Age**	-0.023	-0.029	-0.017	<0.001
**Serum Mg2+**	1.788	0.878	2.698	<0.001
**Constant**	1.490			0.001

EGF: epidermal growth factor; uCr: urinary creatinine, FE Mg^2+^: fractional excretion of magnesium; CI: confidence interval.

#### Urinary EGF

To determine variables that are independently related to urinary EGF, age, creatinine clearance, serum magnesium, serum EGF and the fractional excretion of magnesium were analysed in a multivariate model. As urinary EGF was skewed, a logarithmic transformation was performed and the log uEGF was tested as the dependent variable. Length was not included in the multivariate model since data on length were only available in children. Age serum Mg^2+^ and serum EGF showed to be independently related to urinary EGF (Table 6), while creatinine clearance, and fractional excretion of magnesium were not. The model explains the variation in log uEGF for 56.3%. As we found that gender was also significantly correlated to urinary EGF, we tested this variable in a multivariate model in the population older than 13 years of age. However, gender did not show to be independently correlated to urinary EGF.

**Table 6:** Independent variables related to urinary EGF.

	B	95% confidence interval		p-value
		Lower	Upper	
**Age**	-0.010	-0.015	-0.004	0.001
**Serum Mg^2+^**	0.668	-0.120	1.457	0.096
**Serum EGF**	<0.001	<0.001	0.001	0.003
**Constant**	0.953			<0.001

EGF: epidermal growth factor; CI: confidence interval

## Discussion

This clinical study provides new insights in serum and urinary EGF in children and adults. Firstly, age and length were significantly correlated with both serum and urinary EGF. We therefore propose to use body surface as a correction factor to determine EGF concentrations in children so that EGF values can be interpreted in both children and adults. Similarly, in other biological markers, such as creatinine clearance and cardiac index [29, 30], body surface is used as a correction factor. In children, the bedside Schwartz equation for creatinine clearance is based on serum creatinine and height and expressed as ml/min/1.73m^2^, thereby correcting for body surface [27]. Secondly, in this healthy population, serum EGF was independently related to serum magnesium concentrations. Interestingly, urinary EGF was not related to variables of the magnesium homeostasis.

After correction for body surface, age and serum EGF remained independently and inversely related to each other, with a greater spread in serum EGF values in children. The youngest patients had the highest serum EGF concentrations which exponentially decreased with age. These findings emphasize even more the hypothesis by Joh et al., which suggests that serum EGF is associated with growth and development in children [25]. In addition, urinary EGF/uCr/BSA is inversely and independently related to age, again resulting in a similar exponential curve. Although previous studies observe an inverse correlation between urinary EGF and age [3, 5, 19], we are the first to highlight the exponential curve in this correlation, pointing out the importance of EGF in renal maturation and growth during the early years of life.

Serum EGF/BSA was not related to gender, confirming previous observations by Joh et al. [25]. When considering the role of gender on urinary EGF, however, we statistically titrated a threshold of significance from the age of 13 years on, whereby significantly lower values were noted in men compared to women, meaning gender relates to urinary EGF starting from the age of 13 years, suggesting hormonal influences on renal EGF production. In a study of Mattila in 1986, it was shown that urinary EGF is indeed higher in females older than 20 years [31]. Two other reports, did not find any differences between sexes [24, 25]. They, however, did not divide the study population into age subgroups, thereby possibly missing hormonal influences in the subgroup of puberty.

Serum EGF is positively and independently related to urinary EGF/uCr, indicating that high urinary EGF concentrations accompany high serum EGF concentrations, which contrasts with previous findings [25]. As our multivariate model excludes age as an explanation, we hypothesize that serum EGF might be—at least partially—filtrated by the glomerulus. Although significance was lost in the multivariate model, creatinine clearance was positively related to urinary EGF/uCr/BSA, supporting the hypothesis that serum EGF is glomerularly filtered.

To our knowledge, we are the first to describe a positive relation between serum EGF/BSA and serum magnesium. It would be interesting to further explore this relationship, as this might be a link to the importance of glomerular filtration of EGF in healthy subjects in addition to the previously described tubular origin of urinary EGF [3, 14, 17]. On the contrary, urinary EGF was not associated with serum magnesium neither with the FE Mg^2+^. These findings are new, as in CKD patients, patients after renal transplantation or children with nephrotic syndrome, urinary EGF independently predicts the FE Mg^2+^ [11, 13]. These results might indicate that EGF only becomes an important player in the renal magnesium homeostasis in patients with a chronic kidney condition, possibly also influenced by chronic mediation. Further research is necessary to explore this hypothesis.

This study has limitations. New-borns and children younger 3 were not included. Therefore, this study lacks characteristics specific to this age category. As we only sampled once per patient, we were not able to draw conclusions on causality and therefore report on associations between EGF and biological variables. Still, we were able to investigate correlations with both serum and urinary EGF from both children and adults, allowing us to draw conclusions on normal urinary and serum EGF concentrations in a large age-range. However, in order to provide a genuine set of reference values, another study design should be applied, thereby including intra-subject variability next to the analytical and between subject variability, which would allow for calculating the index of individuality. The EGF values reported in the present study are thus not official reference values in healthy subjects, yet, can be used as indication of normality.

## Conclusion

The insides established in this study on healthy subjects, provide valuable knowledge for comparing and determining the role of EGF in many diseases, such as diabetes and autism spectrum disorder, as well as drug induced conditions. By systematically correcting EGF for body surface, significant correlations with age, gender starting from puberty on and magnesium were observed.
